# Use of fNIRS to Characterize the Neural Mechanism of Inter-Individual Rhythmic Movement Coordination

**DOI:** 10.3389/fphys.2019.00781

**Published:** 2019-07-04

**Authors:** Ruoyu Niu, Yanglan Yu, Yanan Li, Ying Liu

**Affiliations:** ^1^School of Kinesiology, Shanghai University of Sport, Shanghai, China; ^2^Key Lab of Cognitive Evaluation and Regulation in Sport, General Administration of Sport of China, Shanghai, China

**Keywords:** rhythmic movement, movement coordination mechanism, neural mechanism, fNIRS, between individuals

## Abstract

**Background:** Inter-individual rhythmic movement coordination plays an important role in daily life, particularly in competitive sports. Behaviorally, it is more challenging to coordinate alternating movements than symmetrical movements. The neural activity underlying these different movement coordination modes remains to be clarified, particularly considering complex inter-individual coordination differences.

**Methods:** To further test the neural basis of inter-individual rhythmic movement coordination, a revised experimental paradigm of inter-individual coordination was adopted. Participants were asked to perform symmetric, alternate, or single movements (swinging the lower part of the leg) in the same rhythm. A multi-channel, continuous wave, functional near-infrared spectral (fNIRS) imaging instrument was used to monitor hemodynamic activity while 40 volunteers (9 male pairs and 11 female pairs) performed the task. Multivariate analyses of variance were conducted to compare mean oxy-hemoglobin concentration ([HbO]) across experimental conditions.

**Results:** A significant three-way interaction (leg-swing condition × ROI × laterality) on mean [HbO] was observed. *Post hoc* analysis revealed a significant main effect of leg-swing condition only in brain regions of interest [right inferior parietal lobule (IPL)] contralateral to movement execution. Activation in brain regions of interest [right inferior parietal lobule (IPL)] was much stronger in alternate mode compared with symmetric or single modes, and the differences between symmetric and single mode were not statistically significant. This result suggests that the alternate mode of movement coordination was more likely to be supported by the IPL region than the other modes.

**Conclusion:** The present findings provide neural evidence relevant to the theory of self-organization of movement coordination, in which an alternating movement mode appeared to be a more demanding condition than symmetrical movement.

## Introduction

Inter-individual coordinated movement refers to the organized and harmonious movement of body parts as when walking side by side or carrying heavy objects together. For team sports, coordination between 2 or more players is quite normal, as when handing over an object during a relay game or cutting into the restricted area between players and passers during a basketball game (Schmidt et al., 1990). There are two common modes of movement coordination: symmetric and alternate. Symmetric mode is characteristic of teammates working toward a common purpose. In team sports, such as team speed skating and team rowing, synchronization between teammates is common. On the other hand, opposite coordination, as occurs in alternate mode, occurs between opponents. Athletes often orient toward different goals and directions than their opponents, like the offensive batsman and defensive block player in volleyball, and the attack and defense in fencing. The degree of coordination between players can affect competitiveness. Determining the mechanisms of inter-individual coordination modes is important for understanding motor learning and team competitiveness.

Alternate mode is more challenging than symmetric mode for both intra- and inter-individual coordination. Haken et al. (1985) proposed the now classical self-organization theory based on behavioral findings of bimanual rhythmic swing. According to this theory, both symmetric and alternate modes have good stability at low frequencies, and the symmetric mode remains stable at higher frequencies. However, the alternate mode becomes unstable at higher frequencies until the original mode collapses and changes to a symmetric mode (Haken et al., 1985). This theory has accumulated support from a large number of behavioral studies. Bimanual coordination studies have demonstrated instability of the alternate mode, which requires substantial attention to sustain a dynamic system (Temprado et al., 1999; Whitall et al., 1999; Monno et al., 2000; Zanone et al., 2001). Patients with dyskinesia (as occurs in Parkinson disease) have particular difficulty and are prone to errors when performing alternate-mode activities. This theory, originally based on findings from intra-individual coordination, has proven to be equally applicable to inter-individual coordination (Schmidt et al., 1990).

Differences in neuronal activation in different inter-individual coordination modes have yet to be experimentally verified directly. An important characteristic of inter-individual coordination is that the realization of coordination depends on spatially separated brains. Therefore, a crucial neural mechanism for the formation of inter-individual coordinated movements may involve mirror neurons (Schmidt et al., 2011), which localizes to the sectors [anteriorly in inferior frontal gyrus and premotor cortex, posteriorly in the inferior parietal lobule (IPL)] using fMRI, electroencephalogram, and other neuroimaging studies (Rizzolatti et al., 1996; Iacoboni, 1999; Iacoboni et al., 2005; Buccino et al., 2010; Fogassi, 2011). The system provides a basis for the prediction of other persons’ movement intentions (Sebanz and Knoblich, 2010). Such predictions are useful not only for producing imitative actions but also for more complex complementary actions that require spatial and temporal coordination with others (Schmidt et al., 2011).

fNIRS, a relatively new imaging technique, is non-invasive, highly flexible, and robust against movement artifacts. In inter-individual movement coordination, partners interact without severe movement restrictions and with directly observable action sequences. Neural activity during inter-individual coordination of human movements can be observed directly with fNIRS. Thus, in the current study, fNIRS signals were collected while subjects performed different modes of movement coordination. For the present study, we used an experimental inter-individual coordination paradigm that was modified from a prior paradigm (Schmidt et al., 1990). In the synchronized rhythmic leg swing experiments, two participants observe each other’s swinging leg while attempting to coordinate their movements. The relative phases between swinging legs become symmetric (phase angle, 0°) or alternate (phase angle, 180°) over time (Schmidt et al., 1990) upon achievement of spatial and temporal coordination. In Schmidt and colleagues’ study, eight different rhythm frequencies (in Hz: 0.6, 0.8, 1.0, 1.2, 1.4, 1.6, 1.8, and 2.0) were employed to explore the influence of frequency on movement coordination in symmetric and alternate modes; they showed that both modes were stable under the three lowest rhythm frequencies. In the current experimental protocol, we assessed the neural mechanisms of the different coordination modes with a single frequency (see also Wilson et al., 2014). In our low frequency condition (1 Hz), the participants achieved symmetric and alternate coordination easily with stability and little variation. Considering that movement coordination in many sports relies primarily on peripheral vision (Vater et al., 2016), the current experiment was adjusted accordingly. In contrast to the movement observations based on central vision used previously (Schmidt et al., 1990), the current research required that participants use their peripheral vision to fulfill the movement coordination. Additionally, mirror neurons had right laterality (Uddin et al., 2006; Fogassi, 2011) and right frontoparietal area was the focus. This setting leads to the fact that collected brain activities were ipsilateral to action execution for half of the participants and contralateral to action execution for half of participants. This arrangement allows us to examine the relationship between movement coordination and laterality (recording brain area and action execution) simultaneously. All subjects were able to conduct the coordination tasks with competence easily obviating the need to analyze behavioral performance *per se* (see Wilson et al., 2014).

To sum up, to explore the neural basis of inter-individual coordination of rhythmic movements, a modified version of Schmidt et al.’s (1990) paradigm was adopted. Participants were asked to perform symmetric, alternate, or single movements in the same rhythm. Meanwhile, fNIRS was used to explore differences in brain blood oxygen activity for various conditions. Our hemodynamic analysis targeted frontal and parietal areas because previous research has implicated these regions in rhythmic movement coordination. Multivariate analyses of variance (ANOVAs) were used to compare oxygenated hemoglobin concentration ([HbO]) values across experimental conditions. The cognitive processes that may be mediated by these brain regions are addressed in the Discussion section together with how the findings complement existing theory, the shortcomings of this work, and future prospects of this work.

## Materials and Methods

### Participants

Forty right-handed volunteers from Shanghai University of Sports (18 males and 22 females) with a mean age of 22.00 ± 2.10 years with normal or corrected-to-normal vision participated in the study. No participant had a history of physical disability or mental illness. To avoid the potential influence of partner gender (Cheng et al., 2015), participants were paired with partners of the same sex, forming a total of nine male pairs and eleven female pairs. Participants provided informed written consent and were paid for their participation (30¥/h per subject). The study followed the ethical guidelines of the Declaration of Helsinki and was approved by the local ethics committee at Shanghai University of Sports in China (tracking number: 2018010).

### Tasks and Procedures

Each participant sat on a 1-m tall stool located approximately 1.5 m to the side of his or her partner, facing the same direction. Participants observed one another’s limb movements using peripheral vision throughout the experiment. Each participant swung the lower part of his or her outer leg. The stools were modified with a padded plank of wood that protruded 10 cm from the outside corner of the stool to support the leg underneath the knee joint. This setup allowed for comfortable and free movement in the anterior/posterior plane for the lower part of the designated leg — the left leg for the person seated on the left and the right leg for the person seated on the right. A metronome tape was set to a frequency of 1 Hz and used over a 5-s period to serve as a guide for leg swing frequency of participants.

Inter-individual coordination was briefly described to participants. Next, the researcher demonstrated the limb movement. Participants were then asked to swing the appropriate leg at the frequency indicated by the metronome pulse and consciously coordinate their movements in either symmetric or alternate phase modes relative to the other person’s movements. Symmetric mode was described to them as coordination in which their limbs were at the same place in a cycle at the same time. Alternate mode was described as coordination in which their limbs were at opposite places in a cycle at the same time. The two modes were also demonstrated by hand. After this, some practice trials were conducted to familiarize participants with the task.

The experiment used a randomized block design. In a single mode, participants were asked to finish 16 block limb movements alone at the designated frequency. All participant pairs were asked to coordinate their movements in 2 phase modes: eight blocks with symmetric mode and eight blocks with alternate mode. Task conditions were randomly arranged. There was a 30-s rest between blocks.

### Hemodynamic Data Acquisition

**FIGURE 1:**
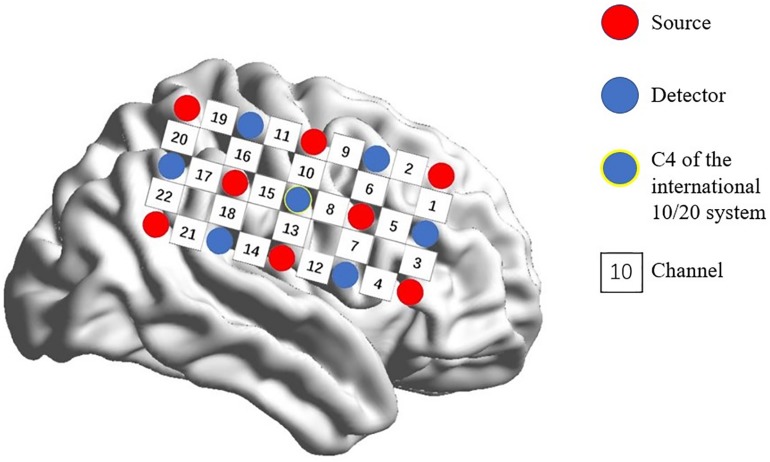
Spatial profile of fNIRS probes. Red circles indicate the eight optical sources. Green circles indicate the seven detectors. Black numbers (1–22) indicate fNIRS channels. The optical sources and detectors were positioned on the international 10–20 standard positions.

A multi-channel, continuous wave, fNIRS instrument (NIRScout; NIRx Medical Technologies LLC; Minneapolis, MN, United States) was used to monitor hemodynamic activity during the task and at rest. The sampling rate was 7.81 Hz. Probes were arranged according to a 10/20 electroencephalogram system with some adjustments to ensure that each emitter was 3 cm from its corresponding detector. Two 3 × 5 optode probe sets (eight emitters and seven detectors, with a 3-cm optode separation) were used. Each set consisted of 22 measurement channels that were placed over the right frontoparietal regions of the brain. The center of the middle probe set row was placed at C4 according to the 10/20 international system (Jurcak et al., 2007; Pan et al., 2017; Figure 1).

### Hemodynamic Imaging

#### Individual-Level Analysis

Because oxygenated hemoglobin signals have a better signal-to-noise ratio than deoxygenated hemoglobin signals, only HbO data were used (Hai-Jing et al., 2013; Schaeffer et al., 2014). The [HbO] data were analyzed in HomER2 (MGH-Martinos Center for Biomedical Imaging; Boston, MA, United States) (Huppert et al., 2009) based on MATLAB (Mathworks; Natick, MA, United States). First, the signal quality of the individual channels was checked by means of a coefficient of variation, which varied from 2 to 47% for all channels. The exclusion value was set at 15% (Piper et al., 2014). By means of a movement artifact reduction algorithm, sampling points greater than 3 standard deviations from the mean were detected and rejected for movement correction (Scholkmann et al., 2010). Subsequently, data were subjected to baseline correction (2–0 s before trial onset) and then bandpass filtered by a high-pass filter with a cutoff frequency of 0.01 Hz to remove low-frequency noise, such as head-movement and by a low-pass filter with a cutoff frequency of 0.15 Hz to attenuate high-frequency noise and cardiovascular artifacts (Huppert et al., 2009; Yunjie et al., 2012). Optical data were converted into hemoglobin signals with mol/L unit in accordance with the modified Beer–Lambert Law (Cope et al., 1987).

#### Group-Level Analysis

In accordance with prior studies (Schultz et al., 2003; Rzucidlo et al., 2013; Alba-Ferrara et al., 2016), we focused on the most activated channels where significant differences were most likely to be found. ROI was defined as channels with maximal [HbO]s. After averaging [HbO]s across participants and experimental conditions, mean [HbO] values for each channel were arranged in descending order, and the top 20% of channels (with greatest values) were defined as channels of interest. The multi-channel fNIRS space was converted into traditional Montreal Neurological Institute coordinate space (Tsuzuki et al., 2007). These channels of interest corresponded to 2 ROI. ROI-1 (channels 5 and 6) was located in the right frontal eye fields and ROI-2 (channels 13 and 14) was located in the right IPL. Subsequently, to reduce signal variation, mean [HbO]s for each ROI (averaged across channels) during the task period were calculated for each experimental condition and subjected to a 2 (ROI: 1 vs. 2) × 2 (laterality: contralateral vs. ipsilateral) × 3 (leg-swing condition: single mode, symmetric mode, and alternate mode) analysis of variance (ANOVA) in SPSS 22.0 (IBM, New York, NY, United States) with ROI and leg-swing condition as repeated measure factors and laterality as a between-subject factor. *Post hoc* analysis (least significant difference) was used to detect the source comparison for observed variance. Mean [HbO] values are reported with standard errors.

## Results

**FIGURE 2:**
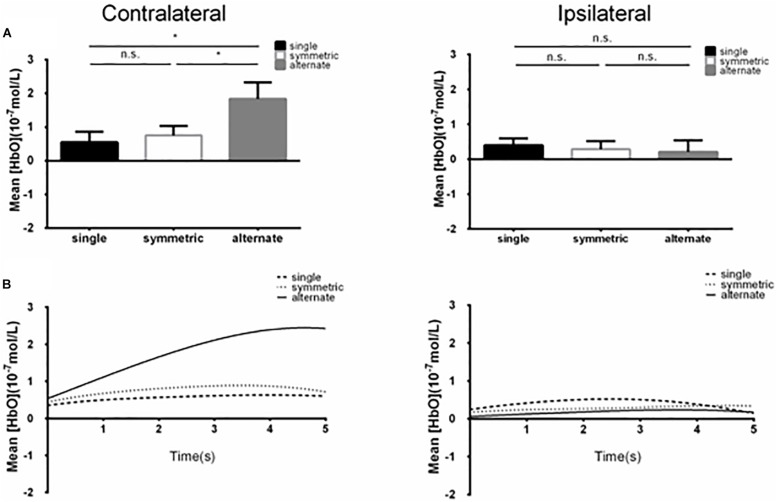
**(A)** Summary of mean [HbO] for the side of the brain contralateral (left) and ipsilateral (right) to movement averaged across ROI-2 (channels 13 and 14) corresponding subjects, and times. Left, the mean [HbO] values for the alternate mode were significantly larger than those obtained for symmetric and single modes; mean [HbO] values did not differ significantly between the symmetric and single modes. ^*^p < 0.05; n.s., *p* > 0.10. Error bar: standard error **(B)** [HbO] time course contralateral (left) and ipsilateral (right) to movement. Mean [HbO] values across two channels (ROI-2, channels 13 and 14) and corresponding subjects are shown.

**FIGURE 3:**
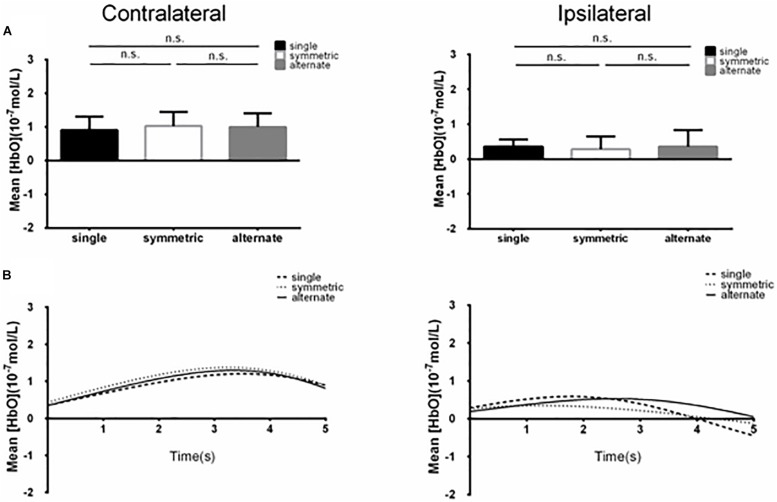
**(A)** Summary of mean [HbO] for the side of the brain contralateral (left) and ipsilateral (right) to movement averaged across ROI-1 (channels 5 and 6) corresponding subjects, and times. n.s., *p* > 0.10. Error bar: standard error **(B)** [HbO] time course contralateral (left) and ipsilateral (right) to movement. Mean [HbO] values across two channels (ROI-1, channels 5 and 6) and corresponding subjects are shown.

A 2 (ROI: 1 vs. 2) × 2 (laterality: contralateral vs. ipsilateral) × 3 (leg-swing condition: single mode, symmetric mode, and alternate mode) ANOVA for mean [HbO] revealed a significant main effect of laterality (*F*_1,38_ = 4.220, *p* = 0.047, partial η^2^ = 0.100). During movement execution, [HbO]s in the contralateral cortex (1.01 × 10^-7^ ± 2.40 × 10^-8^ mol/L) were greater than [HbO]s in the ipsilateral cortex (3.15 × 10^-8^ ± 2.40 × 10^-8^ mol/L). More importantly, the three-way interaction was statistically significant (ROI × laterality × leg-swing condition, *F*_2,76_ = 3.402, *p* = 0.038, partial η^2^ = 0.082). *Post hoc* analysis (least significant difference) showed that only in ROI-2 [right inferior parietal lobule (IPL)] contralateral to movement execution, the mean [HbO] for the alternate mode was significantly higher than that for symmetric mode (*p* = 0.021) and single mode (*p* = 0.003); three was no significant difference between the mean [HbO]s for the symmetric and single modes (*p* = 0.407) (Means and Standard Errors were shown in Table 1; all *P* values from *post hoc* analysis were shown in Table 2; see also Figures 2, 3 for the bar graphs and time courses). Therefore, the null hypothesis of this main effect, which predicted that mean [HbO]s would be equal across the three leg-swing conditions, was rejected. Besides, there was not any significant main effects (leg-swing, *F*_2,76_ = 0.879, *p* = 0.420, partial η^2^ = 0.023; ROI, *F*_1,38_ = 0.008, *p* = 0.929, partial η^2^ = 0.00021) or any significant interactions (ROI × laterality, *F*_1,38_ = 0.079, *p* = 0.780, partial η^2^ = 0.002; ROI × leg-swing condition, *F*_2,76_ = 1.884, *p* = 0.159, partial η^2^ = 0.047; laterality × leg-swing, *F*_2,76_ = 1.333, *p* = 0.270, partial η^2^ = 0.034).

**TABLE 1:** Mean ± Standard Error for each condition (×10^–7^ mol/L).

	**Ipsilateral**		**Contralateral**	
	**ROI-1**	**ROI-2**	**ROI-1**	**ROI-2**
Single mode	0.357 ± 0.320	0.398 ± 0.261	0.907 ± 0.320	0.548 ± 0.261
Symmeric mode	0.282 ± 0.394	0.285 ± 0.256	1.032 ± 0.394	0.752 ± 0.256
Alternate mode	0.355 ± 0.444	0.210 ± 0.419	1.000 ± 0.444	1.834 ± 0.419

**TABLE 2:** P values from *post hoc* analysis (least significant difference) for the 3-way interaction (ROI × laterality × leg-swing condition).

	**Ipsilateral**		**Contralateral**	
	**ROI-1**	**ROI-2**	**ROI-1**	**ROI-2**
Single vs. symmetric	0.811	0.645	0.688	0.407
Single vs. alternate	0.997	0.645	0.834	0.003
Symmetric vs. alternate	0.887	0.867	0.951	0.021

## Discussion

Interpersonal movement coordination plays an important role in daily life, especially in competitive sports. The current study used a rhythmic leg-swing task with three coordination modes (symmetric, alternate, and single). The neural mechanism mediating inter-individual movement coordination was explored with fNIRS. The current fNIRS study showed that the alternate mode produced higher hemodynamic responses in the IPL contralateral to movement execution than did the symmetric and single leg-swing modes. There was no difference between the symmetric and single leg-swing conditions. Based on Irani’s theory, an increase in blood [HbO] in active brain areas indicates increased neural activity in that area (Irani et al., 2007). Accordingly, the neural activation seen with alternate leg-swinging in present study may place the greatest demand on motor imitation (IPL, mirror nervous system). Correspondingly, the present findings provide neurological data applicable to explaining self-organization theory.

The target region was defined based on the obtained [HbO] values. 2 ROI with the highest [HbO] were identified: ROI-1 (right frontal eye fields) and ROI-2 (right inferior parietal lobule). For ROI-2, the alternate mode had a higher [HbO] than the symmetric or single modes. The target region in the current study was consistent with that studied previously.

In the present study, we obtained data linking alternating-movement coordination to right IPL activity as we had expected. Unlike intra-individual coordination, inter-individual coordination requires observation of other people’s actions to identify their intentions and imitate their actions. Thus, inter-individual coordination may involve engagement of mirror neurons. Our findings suggest that the IPL, an important node of mirror neural processing (Rizzolatti et al., 1996; Iacoboni, 1999; Iacoboni et al., 2005; Buccino et al., 2010; Fogassi, 2011) has direct involvement in inter-individual coordination.

Significant differences between modes were obtained only in the cortex contralateral to movement execution, consistent with contralateral control of movement coordination, which would be similar to the contralateral domination of action execution (Kim et al., 1993; Hayashi et al., 2008; Bai et al., 2016).

Current findings have enriched movement coordination related theory on a neurological level. A self-organization theory (Haken et al., 1985) that accounts for behavioral findings (Schmidt et al., 1990) was proposed for these movements. Alternate leg-swing mode was rather unstable (leading to greater standard deviations for the relative phase) at higher frequencies (1.4 Hz, 1.6 Hz, and 2.0 Hz), relative to that at a low frequency (1.0 Hz). Hence, in the unsteady state, there was a tendency for alternate mode to revert to symmetric mode (Schmidt et al., 1990). Alternate mode, therefore, can be inferred to be more complex and require more cognitive resources for inter-individual coordination when compared with symmetric mode. In particular, employing fNIRS in our current research, we were able to make direct observations of differences in brain activity. The alternating mode aroused activation in the IPL, while the other modes were much less effective at doing so. Therefore, the current work provides direct neuroimaging evidence in support of self-organization theory.

This study had two noteworthy limitations. First, to improve the ecological validity of the experiment, we examined peripheral vision instead of central vision, as in the classical paradigm. This change limits our comparisons with previous results from studies that used the traditional paradigm. Second, we employed a single low frequency, which limited the richness of the experimental results. In the future, different frequencies should be examined to further test the neural basis of inter-individual movement coordination. Additionally, different sex-pairings could also be taken into account to verify if gender actually influences inter-individual coordination in a study with a larger sample size designed to test directly gender influences on movement coordination. Recently, methodological progress has been made toward the development of a brain-computer interface with fNIRS (Naseer et al., 2014; Scholkmann et al., 2014; Naseer and Hong, 2015; Nguyen et al., 2016), which enables exploration of superficial brain activity signals that may be used to control an external device during naturalistic behavior. The current findings on the neural mechanism underlying rhythmic inter-individual movement coordination may support the development of an fNIRS-based online brain-computer interface framework to help patients with dyskinesia. In particular, the current findings provide information relevant for identifying indicators for monitoring neuroplasticity in response to neurorehabilitation and neurostimulation.

In summary, inter-individual movement coordination involves activity in the IPL, and activation in these regions is much stronger in the alternate condition. Consistent with self-organization theory, alternate mode was a much more demanding condition than symmetric mode.

## Ethics Statement

Participants provided informed written consent and were paid for their participation. The study followed the ethical guidelines of the Declaration of Helsinki and was approved by the local ethics committee at Shanghai University of Sports in China.

## Author Contributions

YiL and RN designed the experiments. All authors conducted the experiments and analyzed the data. YiL and RN wrote the article.

## Conflict of Interest Statement

The authors declare that the research was conducted in the absence of any commercial or financial relationships that could be construed as a potential conflict of interest.
